# Pseudonyms of Early Tuberculosis

**Published:** 1916-03

**Authors:** 


**Pseudonyms of Early Tuberculosis.** F. H. Smith, Abingdon, Va., Old Dominion Jour. of Med. & Surg., Nov. 1915, 157 cases, 7 of which were probably not tuberculous. Of the 150, 18 were non-pulmonary. The 132 cases of pulmonary tuberculosis remaining had been diagnosed previously as follows: bronchial or catarrhal trouble, 49; stomach trouble 29; weakness, nervousness, etc., 35; indigestion and nervousness in 6; headache or backache or both in 9; miscellaneous diagnoses in 4.

